# Impact of an Updated Climate Database on Model Performance and Niche Variability in Species Distribution Modeling

**DOI:** 10.1002/ece3.73891

**Published:** 2026-06-23

**Authors:** Jae‐Woo Song, Sunhee Yoon, Sunghoon Jung, Wang‐Hee Lee

**Affiliations:** ^1^ Department of Smart Agriculture Systems Machinery Engineering Chungnam National University Daejeon Korea; ^2^ Department of Smart Agriculture Systems Chungnam National University Daejeon Korea; ^3^ Department of Applied Biology Chungnam National University Daejeon Korea

**Keywords:** climate, CLIMEX, database update, MaxEnt, potential distribution

## Abstract

Despite the continuous accrual of occurrence data, the climate datasets used in species distribution modeling (SDM), particularly in mechanistic frameworks such as CLIMEX, often remain based on outdated climate baselines. Here, we present an updated global climate database (1992–2021) and corresponding bioclimatic variables, specifically structured for CLIMEX compatibility while also supporting application across multiple SDM frameworks. By providing a temporally updated climate baseline and enhancing cross‐framework usability, this resource addresses a key barrier to the integration of contemporary climate data into applied ecological modeling. To evaluate its utility, we developed CLIMEX and MaxEnt models for three species and compared model performance, predicted distributions, and niche similarity between the traditional 1961–1990 baseline and the updated 1992–2021 climate data. While overall model performance and broad niche overlaps remained similar between the two periods, localized habitat suitability and occurrence probabilities exhibited noteworthy variability. These results highlight the importance of temporally synchronized climate and occurrence data for reducing predictive uncertainty and accurately assessing climate impacts on species distributions. We provide the updated climate database and associated processing workflows through an open‐access repository. By facilitating the use of contemporary climate data across both mechanistic and correlative SDM approaches, this resource supports reliable pest risk analyses, targeted surveillance, and evidence‐based biosecurity decision‐making under current environmental conditions.

## Graphical abstract

This study introduces an updated open‐access climate database (1992–2021) to replace outdated baselines in species distribution modeling, specifically for CLIMEX and MaxEnt applications. By synchronizing contemporary climate data with recent species occurrences, the researchers demonstrate how to reduce predictive uncertainty and capture localized shifts in habitat suitability. This resource empowers practitioners to conduct more accurate pest risk analyses and formulate biosecurity strategies that reflect current environmental realities.

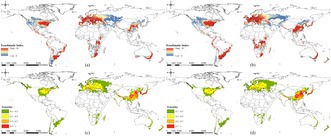

## 1 Introduction

Climate change is a major concern, as it has a considerable impact on life on Earth (Lee et al. 2023). One of the most serious issues caused by climate change is the shift in species habitat, especially for invertebrates, as invasive species have disrupted local ecosystems (Mainka and Howard 2010; Bellard et al. 2012; Halsch et al. 2021). For this reason, the evaluation of the potential distribution of species to prevent negative effects is emphasized, accelerating the application of species distribution models (SDMs) that can predict the potential distribution of a species as a function of abiotic factors (Peterson and Soberón 2012; Sutherst 2014; Byeon et al. 2018).

As climate is a predominant factor determining the habitats of invertebrate species, it has been used as a main variable in SDMs (Karuppaiah and Sujayanad 2012; Kriticos et al. 2015; Skendžić et al. 2021). In other words, meteorological data should be obtained to develop SDMs with a suitable resolution and time span covering the occurrence record period. For example, resolutions of up to 18 km are sufficient for global evaluation, whereas a resolution of at least 1 km is necessary for local predictions (Cornelissen et al. 2019; Jung et al. 2021; Byeon and Lee 2022; Lee et al. 2022; Kanes et al. 2025). Because temporal mismatches between climate data and occurrence records may contribute to uncertainty in SDM predictions, aligning climate datasets with the occurrence record period and incorporating more recent data when available may help improve model reliability. In addition, the required climate data format differs according to the SDM algorithm type, suggesting that raw climate data must be converted to a suitable format for a specific SDM. For instance, MaxEnt uses raster‐based environmental layers (e.g., ASCII format bioclimatic variables), whereas CLIMEX requires climate data to be converted into a proprietary mm file containing monthly meteorological records linked to geographic coordinates. This difference reflects the distinct workflows of correlative and mechanistic SDMs, respectively (Phillips and Dudík 2008; Kriticos et al. 2015).

Owing to the substantial contribution of climate data to SDMs, it is important to secure appropriate types of climate data to develop a reliable model. Fortunately, there are a few public databases which provide climatology specialized for SDMs, such as WorldClim (https://www.worldclim.org), CliMond (https://www.climond.org), and CHELSA (https://chelsa‐climate.org). These sources provide a variety of climate data in terms of resolution, timespan, and file formats. WorldClim and CHELSA freely provide recent high‐resolution meteorological and bioclimatic data that are directly applicable to correlative SDMs (Fick and Hijmans 2017). CliMond is a climatology database for bioclimatic modeling and uniquely provides a suitable format of climate data for CLIMEX modeling (Kriticos et al. 2012). In addition to these public databases, many climate products provide meteorological information suitable for ecological applications. However, differences in temporal coverage, data structure, and model compatibility can limit their direct application across different SDM frameworks, particularly for mechanistic models such as CLIMEX. Moreover, updating climate data is a laborious, slower process than increasing the use of SDMs. In particular, it is necessary to develop climate data applicable to the CLIMEX model, because the data provided by CliMond are outdated.

While SDM is widely recognized as a foundational tool for agricultural and forestry biosecurity, a critical barrier remains in the effective integration of contemporary climate knowledge into operational decision‐making. Historically, updating baseline climate data (e.g., transitioning from the 1961–1990 baseline to recent decades) has presented significant technical and computational barriers for ecologists and policymakers. The raw meteorological data required for these updates are often formatted in complex arrays that are computationally intensive to downscale and process into biologically meaningful variables. Consequently, practitioners often rely on outdated, yet easily accessible, historical climate baselines for current pest risk analyses, which compromises the reliability of predictive models. To address this practical limitation, this study aimed to develop standardized climate databases from publicly available meteorological data and evaluate their applicability using representative pest species. These databases can be adapted to different types of SDMs, including correlative and mechanistic models. Specifically, we developed a globally processed, CLIMEX‐compatible meteorological database representing the 30‐year climate normal for 1992–2021 using publicly available climate data sources. The database provides a temporally updated alternative to the meteorological datasets in MetManager (*.mm) format traditionally used in CLIMEX. We also derived 19 bioclimatic variables from the same 1992–2021 baseline to support MaxEnt and other correlative SDMs. Subsequently, we used these newly developed databases to assess the potential distribution of pests and compared the results with those of models based on existing climate databases.

## 2 Materials and Methods

### 2.1 Meteorological Data Acquisition

Basic meteorological data for constructing the most recent climate database for SDM operations were obtained from WorldClim (https://www.worldclim.org/) (Fick and Hijmans 2017; Harris et al. 2020). The data recorded average monthly maximum temperature (tmax), monthly minimum temperature (tmin), and monthly precipitation (prec) from 1992 to 2021 at 18.5 km. The obtained data were in raster format and saved as a tag image file (TIFF) to record climate data. To obtain a suitable format for the climate database required by CLIMEX, MaxEnt, and other correlative SDMs, we spatially processed the raw data using ArcGIS (version 10.4.1; ESRI, Redlands, USA).

### 2.2 Meteorological Data Processing for mm File and Bioclimatic Variables

**FIGURE 1:**
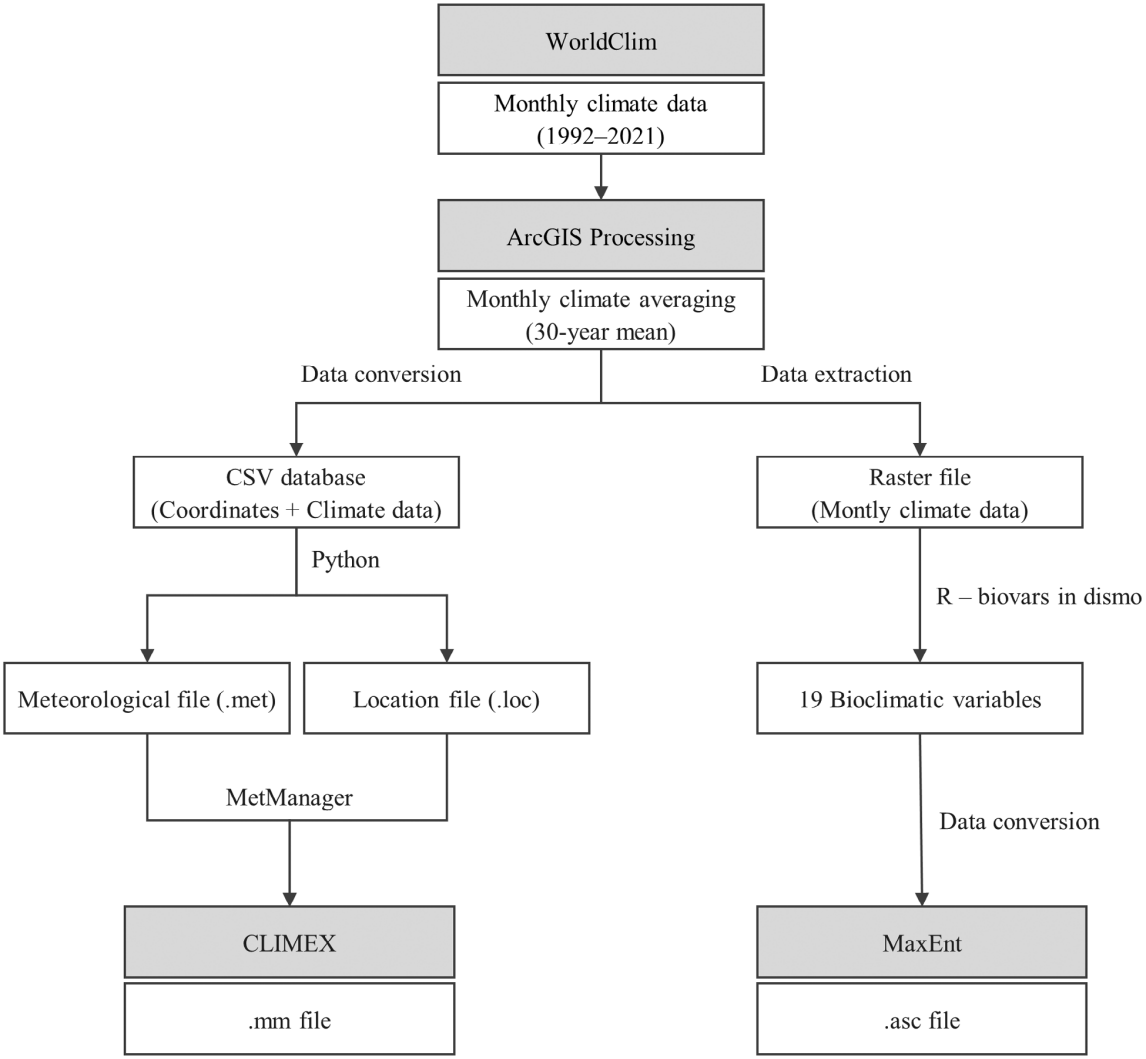
Workflow for constructing climate databases compatible with mechanistic (CLIMEX) and correlative species distribution models.

The acquired global climate data were processed to construct climate databases compatible with either mechanistic (CLIMEX) or correlative (MaxEnt) species distribution models (Figure 1). First, the pixels in the raster file were converted into points, and all points were georeferenced using the World Geodetic System (WGS) 1984 coordinate system. This conversion was necessary because MetManager requires climate information to be organized as point‐based records rather than raster layers. The file was then saved as a comma‐separated values (CSV) file composed of columns and rows coding coordinated points and monthly tmax, tmin, and prec. To facilitate parameter fitting during CLIMEX modeling, 578,888 rows were divided into two groups using ArcGIS: 98,888 rows from the less influential northern part and 480,000 rows from the remaining southern part. This partitioning was necessary because MetManager occasionally generated processing errors when handling very large input datasets. The partitioning was performed solely for computational purposes and did not alter the climatic information contained in the final database, as both subsets were subsequently combined and used together in CLIMEX analyses.

Based on a global CSV file containing climate information for each month (January to December) and latitude and longitude information for each point, a simple Python code was used to convert it into met (meteorological data) and loc (location data) files according to the formats found in the CLIMEX User's Guide (Kriticos et al. 2015; Python 2021). Monthly climate rasters were first processed in ArcGIS to extract grid‐cell coordinates and associated climate variables. The resulting tables were merged into a standardized CSV file, which was subsequently converted into CLIMEX‐compatible .met and .loc files using a custom Python script (Code S1). The met files were structured in the following order: monthly maximum temperature, minimum temperature, precipitation, relative humidity (9 a.m. and 3 p.m.), and location (latitude and longitude). However, the relative humidity was not used because it is not directly required for the operation of the CLIMEX model and is difficult to obtain (not provided by WorldClim). The locations (latitude and longitude) in the met file were coded in the same order and with the same names as in the loc file, which consisted of the continent, country, and state or region name, location (latitude and longitude), and altitude. To create a loc file, the latitude and longitude were used up to seven decimal places. There were no duplicate coordinates, and the loc and met files had the same name to construct an mm file (the final format of meteorological data for CLIMEX) using the MetManager function implemented in CLIMEX (Kriticos et al. 2015).

The global climate files (18.5 km resolution) downloaded from WorldClim consisted of 1080 files (12 months for 30 years for three datasets of tmin, tmax, and prec). Using the raster calculator tool in ArcGIS, the average value for 30 years was calculated and exported in TIFF format. The TIFF files were used in the biovars function in the R software ver. 4.2.2 dismo package ver. 1.3‐14 to generate 19 bioclimatic variables, which were then converted to ASCII format for use in a correlative SDM (Hijmans et al. 2017; R Core Team 2021). No variable filtering was applied during this generation step; species‐specific variable selection was subsequently performed during MaxEnt model development based on correlation analysis and biological relevance.

### 2.3 Model Operation

#### 2.3.1 Example Species and Occurrence Data

**FIGURE 2:**
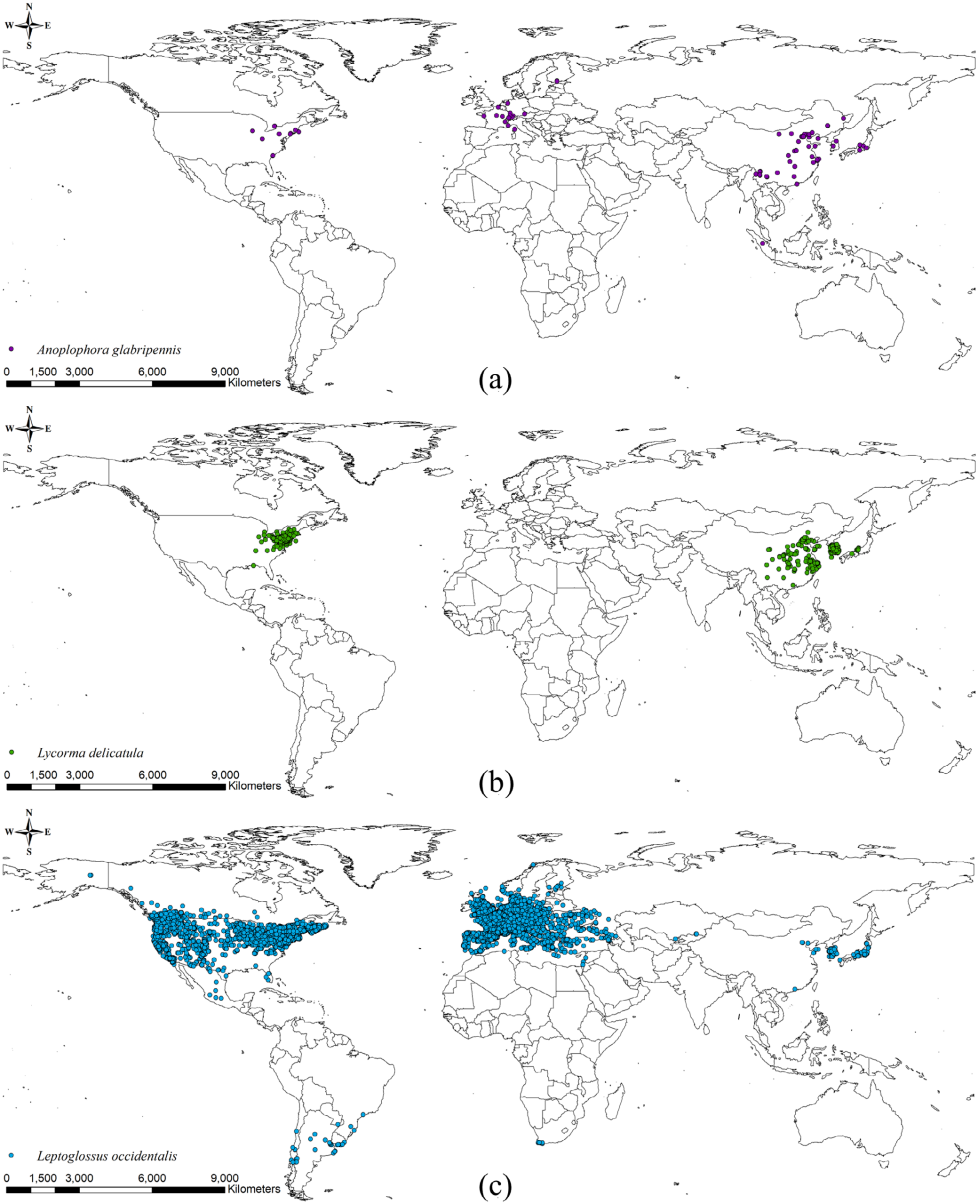
Global occurrence points of (a) *Anoplophora glabripennis* , (b) *Lycorma delicatula*, and (c) *Leptoglossus occidentalis* .

To test the developed database, we selected three pest species: *Anoplophora glabripennis* (Motschulsky), *Lycorma delicatula* (Hemiptera: Fulgoridae), and *Leptoglossus occidentalis* Heidemann (Heteroptera: Coreidae). These species were selected because they are economically important invasive pests that have caused substantial agricultural and forest damage in multiple regions worldwide. In addition, they were chosen based on the availability of sufficient occurrence records and previously published CLIMEX and/or MaxEnt models, which enabled direct comparison between model outputs generated using historical and updated climate databases. The selected species also differ in their current geographic distributions and climatic associations, providing representative case studies for evaluating the applicability of the newly developed climate databases across multiple pest species (Jung et al. 2017; Byeon, Kim, et al. 2021; Byeon, Jung, et al. 2021). Dataset compatibility was verified before model application by checking that the updated climate layers used the same coordinate system, units, global extent, variable definitions, and file formats as the historical datasets used in the original CLIMEX and MaxEnt models. The occurrence data of the three species were collected from multiple sources at the Global Biodiversity Information Facility, Centre for Agriculture and Bioscience International (CABI, https://www.cabi.org), and from previous studies to reduce uncertainty in occurrence records (Jung et al. 2017; Byeon, Kim, et al. 2021; Byeon, Jung, et al. 2021; GBIF 2023a, 2023b, 2023c). Occurrence records were first standardized and cleaned by removing records with missing coordinates, invalid coordinate ranges, and duplicate latitude–longitude pairs. Consequently, occurrence records up to 2022 were obtained and spatially filtered with a 10 km buffer to remove sampling bias, with a total of 80, 874, and 8328 records for *A. glabripennis* , *L. delicatula* , and *L. occidentalis* , respectively (Figure 2). Although different thinning distances may influence SDM outcomes, a 10 km buffer was selected based on its widespread use in previous species distribution modeling studies, including studies on the target species used in this study (Boria et al. 2014; Byeon, Kim, et al. 2021; Yoon et al. 2026).

#### 2.3.2 CLIMEX Operation

CLIMEX (version 4.0, Hearne software, Melbourne, Australia) is a climate‐specialized species distribution model that connects the regional climate to species biology and is coded by model parameters (Kriticos et al. 2015). Model parameters were determined based on experimental results as well as climatic conditions in habitats; then, the parameters were iteratively adjusted until the simulated climatic suitability showed the closest agreement with the known geographical distribution and occurrence records of the target species (Jung et al. 2016). The final outcome of the CLIMEX model is the Ecoclimatic Index (EI) representing the climatic suitability of a target species in a specific area. The EI ranges from 0 to 100, with higher values indicating greater climatic suitability owing to favorable climatic conditions. Definitions of the model parameters and indices have been described elsewhere, and the CLIMEX modeling process has been previously published (Kriticos et al. 2015; Jung et al. 2016, 2017; Byeon, Kim, et al. 2021; Byeon, Jung, et al. 2021). Here, the CLIMEX model was established using previously published parameter values (Jung et al. 2017; Byeon, Kim, et al. 2021; Byeon, Jung, et al. 2021) (Table 1). The CLIMEX model was then operated under the existing climate database recording for 1961–1990 and the currently constructed database for 1992–2021. The EI values from each model were projected onto a world map using ArcGIS (version 10.4.1; ESRI, Redlands, CA, USA).

**TABLE 1:** CLIMEX parameter values for three example species.

Parameters	Code	*A. glabripennis* (Byeon, Kim, et al. 2021)	*L. delicatula* (Jung et al. 2017)	*L. occidentalis* (Byeon, Jung, et al. 2021)
Temperature				
Limiting low temperature (°C)	DV0	6.5	8	13.4
Lower optimal temperature (°C)	DV1	20	16	21
Upper optimal temperature (°C)	DV2	28	30	28
Limiting high temperature (°C)	DV3	33	33	31
Day‐degrees (day)	PDD	1000	355.4	—
Moisture				
Limiting low soil moisture	SM0	0.05	0.3	0.05
Lower optimal soil moisture	SM1	0.2	0.5	0.1
Upper optimal soil moisture	SM2	1.1	1.5	0.8
Limiting high soil moisture	SM3	1.5	2.5	1.4
Cold stress (CS)				
CS temperature threshold (°C)	TTCS	−1.5	0	−10
CS temperature rate	THCS	−0.0002	−0.0005	−0.0005
CS degree‐day threshold (°C)	DTCS	—	—	—
CS degree‐day rate	DHCS	—	—	—
Heat stress (HS)				
HS temperature threshold (°C)	TTHS	29	35	31
HS temperature rate	THHS	0.0015	−0.0005	0.0015
Dry stress (DS)				
DS threshold	SMDS	0.15	0.1	0.05
DS rate	HDS	−0.01	−0.005	−0.005
Wet stress (WS)				
WS threshold	SMWS	1.2	2.5	1.4
WS rate	HWS	0.01	0.002	0.001

#### 2.3.3 MaxEnt Operation

MaxEnt is a correlative species distribution modeling tool that estimates occurrence probability based on environmental characteristics in the occurrence areas of a target species (Phillips and Dudík 2008). This algorithm uses the maximum entropy theory to learn the environmental characteristics of the occurrence area to derive the potential distribution of a species. It is popular because it is a presence‐only model and includes any type of environmental variable with a suitable format. In addition, both the developmental process and necessary tools are well equipped, facilitating convenient utilization of the model. Here, we reproduced the existing MaxEnt models for *A. glabripennis* using the same bioclimatic variables as in a previous study, which used data from 1970 to 2000, whereas the current study used data from 1992 to 2021 (Byeon, Kim, et al. 2021) (Table 2). For *L. delicatula* , and *L. occidentalis* , new MaxEnt models were developed because no previous model was applicable for this study. After extracting the values of the bioclimatic variables corresponding to the occurrence coordinates of these pests, a correlation analysis was performed to remove highly correlated (*r* > 0.7), biologically important variables to minimize spatial autocorrelation (Barta 2016; Lee et al. 2019). Finally, ten and seven bioclimatic variables were selected for *L. delicatula* , and *L. occidentalis* , respectively. Using ENMeval for both models, the optimal model feature and regularization multiplier for both pests were linear + quadratic + hinge + product + threshold and 0.5, respectively (Muscarella et al. 2014). All models were operated with 10,000 randomly selected background points distributed throughout the entire global extent of the climate database and a 10‐fold cross‐validation. The outcomes were stored in logistic format and projected onto a map using ArcGIS software.

**TABLE 2:** MaxEnt model variables for three example species.

Species	Bioclimatic variables	Description	Contribution (%)
*A. glabripennis*	Bio2	Mean diurnal range	9.7
	Bio3	Isothermality	16.2
	Bio5	Max temperature of warmest month	5.7
	Bio6	Min temperature of coldest month	39.2
	Bio12	Annual precipitation	1.5
	Bio15	Precipitation seasonality	5.8
	Bio18	Precipitation of warmest quarter	22.5
*L. delicatula*	Bio2	Mean diurnal range	2.6
	Bio5	Max temperature of warmest month	9.4
	Bio6	Min temperature of coldest month	40.9
	Bio8	Mean temperature of wettest quarter	12.7
	Bio12	Annual precipitation	11.9
	Bio13	Precipitation of wettest month	22.2
	Bio17	Precipitation of driest quarter	0.4
*L. occidentalis*	Bio1	Annual mean temperature	67.8
	Bio2	Mean diurnal range	0.2
	Bio3	Isothermality	7.9
	Bio4	Temperature seasonality	9
	Bio8	Mean temperature of wettest quarter	0.6
	Bio9	Mean temperature of driest quarter	0.3
	Bio10	Mean temperature of warmest quarter	6
	Bio13	Precipitation of wettest month	2.6
	Bio14	Precipitation of driest month	5
	Bio18	Precipitation of warmest quarter	0.7

#### 2.3.4 Model Performance Evaluation

With climate databases, we used True Skill Statistics (TSS) to evaluate model performance because it is known to be the most practical in species distribution modeling (Allouche et al. 2006). True Skill Statistics was calculated using R software and was calculated by adding the sensitivity and specificity calculated based on the model results and subtracting 1 without using any special packages. For CLIMEX outputs, binary suitability maps were derived using the EI threshold that yielded the highest TSS value. TSS was then calculated as a common threshold‐based evaluation metric to ensure a consistent comparison between the two modeling approaches (Yoon and Lee 2023). Sensitivity and specificity were also reported separately as complementary evaluation metrics to improve the comparability of model performance between CLIMEX and MaxEnt. The area under the receiver operating characteristic curve (AUC) was assessed for MaxEnt. For CLIMEX, recall was calculated as the proportion of observed occurrence records falling within climatically suitable areas (EI ≥ 1). This metric has been widely adopted in CLIMEX studies because model parameterization aims to achieve close agreement between predicted climatic suitability and known species distributions (Byeon and Lee 2022; Jung et al. 2023; Yoon and Lee 2023).

## 3 Results

### 3.1 Climate Database

The format of the climate databases was developed using a mm file (.mm) and ASCII format (.asc) for CLIMEX and MaxEnt, respectively. The current global mm file included a total of 578,888 pixels (coordinates) at 18.5 km resolution with a size of 2.05 GB. Bioclimatic variables had the same number of pixels as the mm file and a total size of 365 MB, ranging from 18.2 to 19.6 MB per type of bioclimatic variable.

### 3.2 Evaluation of Model Performance as Applied to Example Species

Overall, all models showed acceptable performance, with TSS values ranging from 0.56 to 0.70 for CLIMEX and from 0.83 to 0.94 for MaxEnt (Table 3). The lowest TSS was observed in the CLIMEX model for *L. delicatula* , whereas the highest TSS was observed in the MaxEnt model for *A. glabripennis* . Sensitivity values were generally high across all models, ranging from 0.86 to 0.95 for CLIMEX and from 0.94 to 1.00 for MaxEnt. Specificity values ranged from 0.63 to 0.80 for CLIMEX and from 0.83 to 0.97 for MaxEnt, indicating that both modeling approaches correctly identified a large proportion of occurrence areas. The performance of the CLIMEX models increased when using the recent climate database, with the largest increment in recall (0.09) for *the A. glabripennis* model. In the MaxEnt model, the recent climate database slightly enhanced the TSS of *the L. delicatula* model; however, the other two models showed lower TSS values than the model with the existing database. In particular, using the latest climate database, the TSS of *A. glabripennis* decreased by 0.11 compared to the model using the previous database. However, AUC values showed only slight differences between climate databases, ranging from 0.95 to 0.96 for *A. glabripennis* , remaining at 0.97 for *L. delicatula* , and decreasing slightly from 0.79 to 0.78 for *L. occidentalis* . The pixel ratio—representing the size of the predicted area where species were expected to occur—was much larger in the CLIMEX model than in the MaxEnt model, and the database types resulted in a small difference of within 2% in predictions for the same species, except for the CLIMEX model for *L. delicatula* (6% difference).

**TABLE 3:** Performance evaluation for CLIMEX and MaxEnt with different climate databases.

CLIMEX						
Species	Climatic database	Sensitivity	Specificity	TSS^a^	Recall^b^	Pixel ratio^c^
*A. glabripennis*	1961–1990	0.86	0.80	0.66	0.87	0.26
	1992–2021	0.95	0.75	0.70	0.96	0.26
*L. delicatula*	1961–1990	0.94	0.63	0.57	0.99	0.35
	1992–2021	0.92	0.67	0.59	0.99	0.29
*L. occidentalis*	1961–1990	0.88	0.68	0.56	0.97	0.43
	1992–2021	0.94	0.66	0.60	0.98	0.43

MaxEnt						
Species	Climatic database	Sensitivity	Specificity	TSS^a^	AUC	Pixel ratio^c^
*A. glabripennis*	1971–2000	1	0.94	0.94	0.96	0.11
	1992–2021	1	0.83	0.83	0.95	0.09
*L. delicatula*	1971–2000	0.94	0.97	0.91	0.97	0.12
	1992–2021	0.97	0.96	0.93	0.97	0.11
*L. occidentalis*	1971–2000	0.9	0.93	0.88	0.79	0.04
	1992–2021	0.96	0.89	0.85	0.78	0.03

^a^ TSS: calculated as sensitivity + specificity −1. Binary suitability maps used for TSS calculation were generated using the threshold that maximized the TSS value for each model.

^b^ Recall: the ratio of the number of actual occurrence points included in the region evaluated as climatically suitable (EI ≥ 1) to the total number of actual occurrence points.

^c^ Pixel ratio: the ratio of the projected number of pixels in which an example species is expected to occur to the total number of pixels in a climate database. The areas in which the example species can occur were produced by converting the result into a binary map with a threshold value that maximizes the TSS.

### 3.3 Global Prediction of Example Species by the Climate Database

**FIGURE 3:**
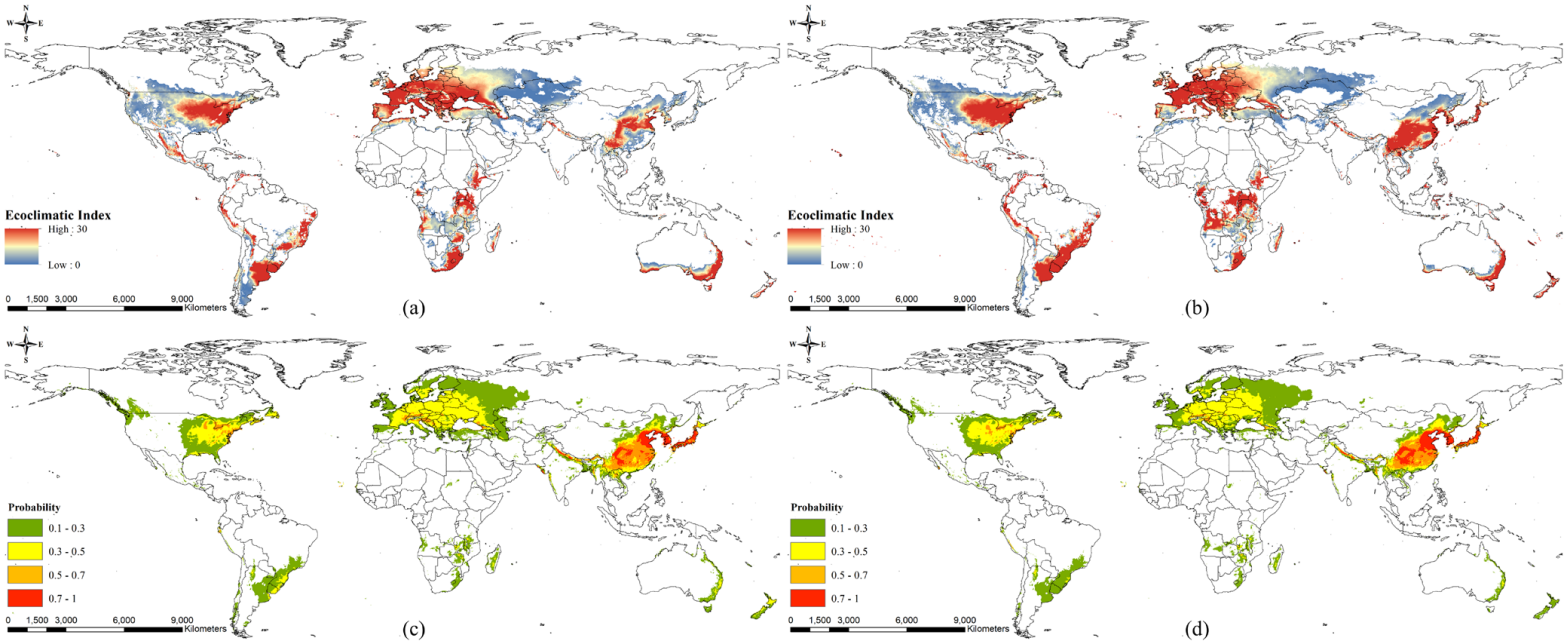
Potential distribution of *A. glabripennis* using (a) CLIMEX with climatic data from 1961 to 1990, (b) CLIMEX with climatic data from 1992 to 2021, (c) MaxEnt with climatic data from 1971 to 2000, and (d) MaxEnt with climatic data from 1992 to 2021.

**FIGURE 4:**
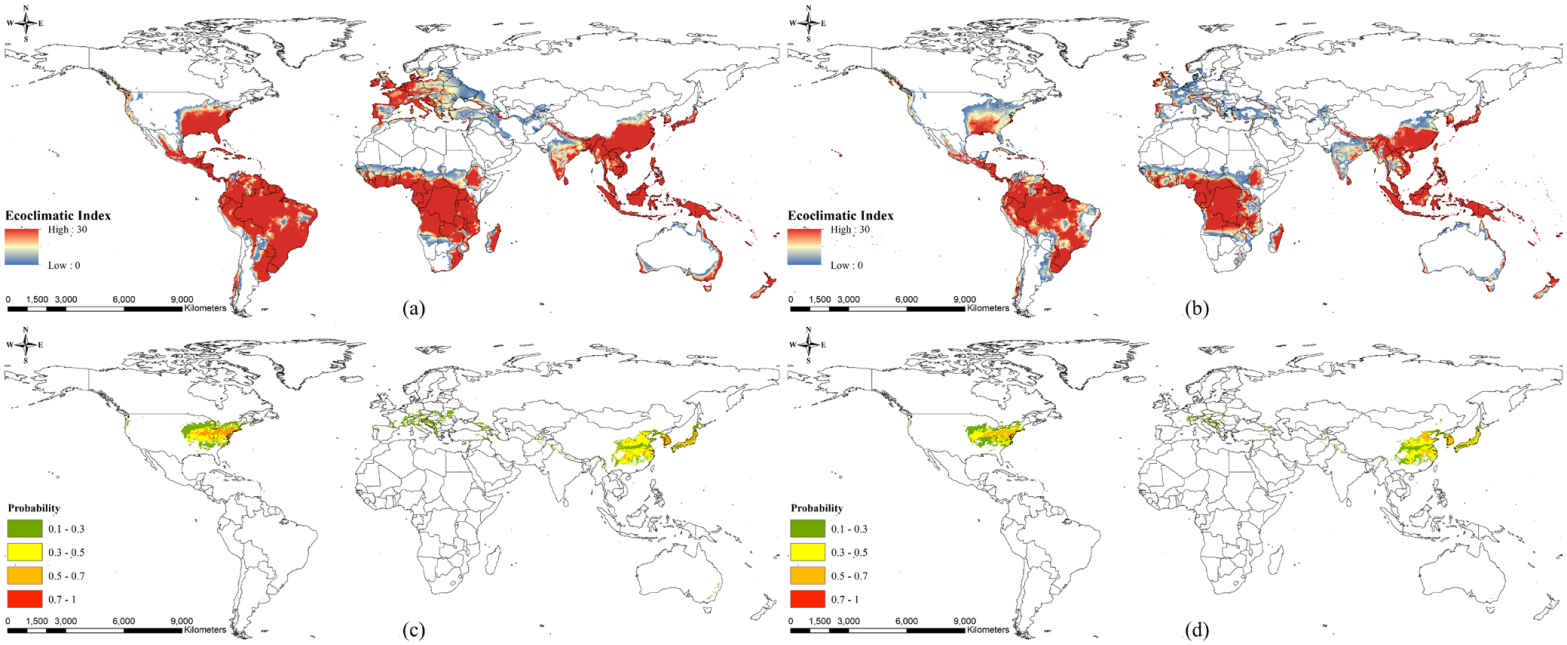
Potential distribution of *L. delicatula* using (a) CLIMEX with climatic data from 1961 to 1990, (b) CLIMEX with climatic data from 1992 to 2021, (c) MaxEnt with climatic data from 1971 to 2000, and (d) MaxEnt with climatic data from 1992 to 2021.

**FIGURE 5:**
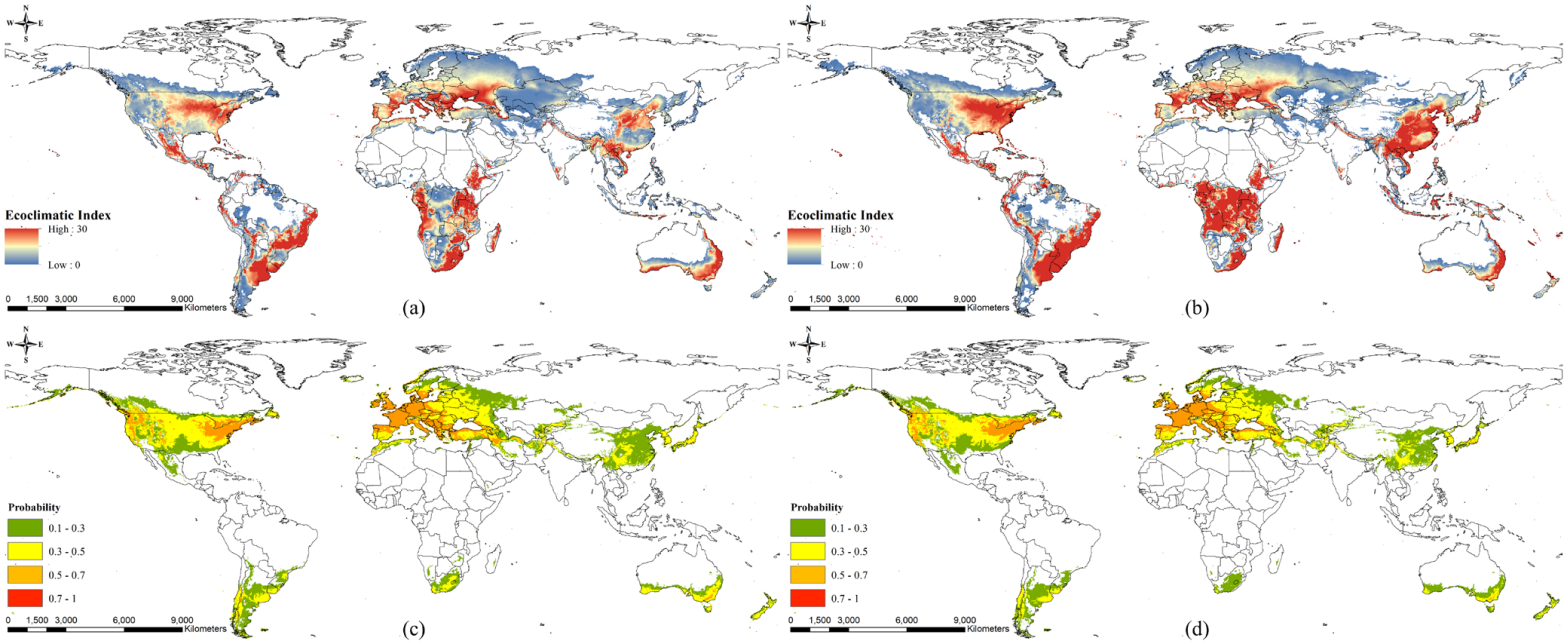
Potential distribution of *L. occidentalis* using (a) CLIMEX with climatic data from 1961 to 1990, (b) CLIMEX with climatic data from 1992 to 2021, (c) MaxEnt with climatic data from 1971 to 2000, and (d) MaxEnt with climatic data from 1992 to 2021.

According to the climate database, there were no significant differences in the predicted potential distribution regions of the pests in both the CLIMEX and MaxEnt models based on Schoener's D and Hellinger's I, which are measures of niche overlap (Warren et al. 2008, 2010; Rödder and Engler 2011) (Table 4). Generally, the distributions predicted by CLIMEX using the historical and updated climate databases showed lower similarity than those predicted by MaxEnt, and the similarity was different for the example species, as well as the metrics of niche overlap. The largest difference was observed in the CLIMEX model for *L. occidentalis* , whereas in the MaxEnt model, *A. glabripennis* was almost identical. Even though a niche overlap similarity was observed, there were differences in the distribution level of climatic suitability and occurrence probability (Table 5). With CLIMEX, there was a marked difference in the number of pixels with an EI > 30, whereas the pixel difference was pronounced with occurrence probabilities > 0.1 in MaxEnt (Figures 3, 4, 5). For example, in the CLIMEX model, the number of pixels in the optimal area with EI values > 30 for *L. occidentalis* more than doubled from 23,557 using the existing climate database to 47,873 using the updated climate database, the largest change among the three species (Table 5). A clear increase in regions with EI values > 30 was observed mainly in the Americas, Africa, and East Asia, excluding Europe (Figure 5). In the case of *A. glabripennis* , the number of pixels with EI values > 30 increased by 37%, which is a lower percentage, but in the case of *L. delicatula* , the number of pixels decreased by approximately 40% (Table 5). For *A. glabripennis* , an increase in areas with EI values > 30 was confirmed in the eastern United States, southern South America, and central Africa, with a particularly marked difference confirmed in East Asia, while a decrease in suitable habitat areas was confirmed in Europe and Australia. In the case of *L. delicatula* , it was confirmed that all areas with EI values > 30 showed an overall decrease, and in particular, a clear difference in suitable habitat areas was confirmed in the United States and Europe. In the MaxEnt model, the number of pixels in areas with occurrence probability > 0.5 decreased by approximately 6% for *A. glabripennis* and approximately 10% for *L. occidentalis* , while the number was almost the same for *L. delicatula* (Table 5). The changes in regions with occurrence probability > 0.5 were not significant in all three models, unlike the CLIMEX model (Figures 3, 4, 5). The overall distribution prediction area was narrower than the results of the CLIMEX model, and in particular, the difference was very large for *L. delicatula* , making it almost impossible to find occurrence probability in South America and Africa (Figures 3, 4, 5). This indicates that climate database updates resulted in differences in habitat suitability within potential occurrence areas rather than across the entire geographical range.

**TABLE 4:** Measurement of niche overlap for three example species of *Anoplophora glabripennis* , *Lycorma delicatula*, and *Leptoglossus occidentalis* .

Species	Model	Schoener's D	Hellinger's I	Average
*A. glabripennis*	CLIMEX	0.712	0.857	0.785
	MaxEnt	0.908	0.935	0.922
*L. delicatula*	CLIMEX	0.785	0.835	0.810
	MaxEnt	0.815	0.959	0.887
*L. occidentalis*	CLIMEX	0.741	0.912	0.827
	MaxEnt	0.919	0.992	0.956

**TABLE 5:** The number of pixels by Ecoclimatic Index in the CLIMEX model according to climate change.

CLIMEX						
Species	Climatic database	EI = 0	0 < EI ≤ 10	10 < EI ≤ 20	20 < EI ≤ 30	EI > 30
*A. glabripennis*	1961–1990	420,467	57,827	32,847	24,992	29,668
	1992–2021	430,259	53,487	29,262	25,006	40,874
*L. delicatula*	1961–1990	369,607	36,271	25,522	23,830	110,571
	1992–2021	409,311	48,987	27,695	25,045	67,850
*L. occidentalis*	1961–1990	323,583	120,440	60,616	37,605	23,557
	1992–2021	328,824	111,281	53,235	37,675	47,873

MaxEnt						
Species	Climatic database	*p* ≤ 0.1	0.1 < *p* ≤ 0.3	0.3 < *p* ≤ 0.5	0.5 < *p* ≤ 0.7	*p* > 0.7
*A. glabripennis*	1971–2000	472,197	60,368	31,754	10,054	4515
	1992–2021	481,718	56,282	27,231	8390	5267
*L. delicatula*	1971–2000	557,367	10,193	8423	2896	9
	1992–2021	561,698	7735	6658	2793	4
*L. occidentalis*	1971–2000	447,494	54,609	53,320	23,465	0
	1992–2021	458,519	53,260	45,910	21,196	3

*Note:* The EI and occurrence probability categories were used to compare changes in the distribution of climatic suitability and occurrence probability between climate databases.

## 4 Discussion

Climate is a paramount factor exerting a considerable influence on the potential distribution of insects, rendering it an indispensable variable in the field of species distribution modeling (Miller 2010; Srivastava et al. 2019). Therefore, to develop a more reliable model, it is necessary to reduce the uncertainty of the climate data (Stoklosa et al. 2015). However, even though continuous updates are applied to occurrence data with the latest information, climate data updates lag behind and are unable to match the timeline of the occurrence data. To provide a more dependable evaluation of the recent distributions of insect species, we need climate variables that match the data collection period of the occurrence data (Elith and Leathwick 2009). Here, there was no significant improvement in model performance with the new climate database and the potential distribution of the example species, despite the time gap in climate data and the use of species occurrence records collected until 2022. The number of occurrence coordinates collected after 1990 or 2000 was larger within or in proximity to the occurrence areas observed before 1990 or 2000 than in regions with significant geographical disparities from the initial occurrence areas. Because model performance depends on the size and distribution of occurrence data, this trend in occurrence data may limit the influence of climate data on model performance (Elith and Graham 2009). Additionally, temporal resolution affects model performance (Pennino et al. 2019). From this perspective, the utilization of a 30‐year average to reduce noise and variation diminishes the influence of climate data variation on species distribution modeling.

Although the potential distribution areas remained relatively consistent according to the climate database, notable variations were observed in the climatic suitability and occurrence probabilities within these regions, underscoring the necessity for climate database updates (Elith and Leathwick 2009). This pattern was further supported by niche similarity metrics. Because Hellinger's I quantifies niche overlap based on occurrence probabilities, whereas Schoener's D is more sensitive to changes in suitability values by assuming proportionality between suitability and abundance (Wellenreuther et al. 2012), the observed combination of high Hellinger's I and low Schoener's D indicates that the overall extent of the potential distribution remained largely unchanged, while suitability values within that distribution underwent shifts. This discrepancy between relatively stable evaluation metrics and noticeable shifts in spatial suitability patterns suggests that climate database updates primarily affected the intensity of climatic suitability within already suitable regions rather than causing large‐scale changes in overall distribution boundaries. The results indicate that SDMs may be relatively insensitive to changes in long‐term baseline climate periods when evaluated using global performance metrics, while still exhibiting substantial local variation in climatic suitability. Such differences are most likely to occur in marginally suitable regions near the limits of species climatic tolerance, where relatively small climatic changes can alter suitability levels and potentially influence species establishment and invasion risk. Furthermore, the consistency in the change patterns observed in models utilizing databases processed in a manner suitable for different algorithms indicates a clear effect of updated climate data, suggesting the reliability of recently constructed climate datasets.

Compared to MaxEnt, CLIMEX showed less similarity between the predicted potential distributions of all example species in all niche overlap metrics. This might be because the characteristics of the CLIMEX model—as a mechanistic model that integrates the biological traits of species and climate—lead to a wider range of suitable habitats than the MaxEnt model, which derives similar regions by learning the environmental characteristics of occurrence areas (Song et al. 2022). Because MaxEnt derives species–environment relationships directly from occurrence data, changes in climate databases may have a smaller influence on model outputs when the environmental conditions associated with occurrence records remain broadly consistent. In contrast, CLIMEX derives growth and stress indicators based on conditions that promote or limit species growth. This multi‐step modeling process may result in greater sensitivity to climate updates compared to algorithms that simply identify environmentally similar regions (Jung et al. 2016). The CLIMEX model has a wider range of EI values than the probability in MaxEnt, possibly a reason for the lower similarity in the CLIMEX model. Furthermore, although it may not yield substantial discrepancies, it is worth noting that the existing CLIMEX database is older than that utilized in MaxEnt in the context of accelerating climate change.

The largest similarity was shown in the MaxEnt model of *L. occidentalis* due to the abundant and widely distributed *L. occidentalis* data resulting in a high prevalence (van Proosdij et al. 2016). The notable performance change was observed in the MaxEnt model for *A. glabripennis* . However, this change likely reflects differences in threshold‐dependent classification outcomes associated with the updated climate database rather than substantial changes in the overall predictive capacity of the model, as AUC values remained largely unchanged between climate databases. This is because a large number of occurrence points across a wide area may result in higher variability in climate values at each coordinate, which, in turn, could lead to a lower impact from changes in climate data. In contrast, *L. delicatula* is concentrated in a narrow geographical area, which could result in lower climate data variability at its occurrence coordinates. Therefore, when climate data are updated, they may exhibit more significant changes, as has been shown for other species. This pattern may also explain why the MaxEnt model predicted more restricted suitable areas for *L. delicatula* in South America and Africa. Because the available occurrence records were concentrated primarily in East Asia and North America, the climatic conditions represented by these records may have been relatively narrow. Accordingly, MaxEnt may have projected regions with similar environmental conditions outside the observed range more conservatively, whereas CLIMEX identified additional suitable areas based on model parameters representing species‐specific responses to climate. This difference reflects the distinction between the fundamental niche and the realized niche represented by the two models. In CLIMEX, species biology is more closely associated with niche similarity than with occurrence data. When a species has a wide climate adaptation range, climate data updates may result in minimal effects; however, for climate‐sensitive species, changes stemming from climate data can be considerable. In the CLIMEX model, climatic suitability is rated lower, with a high stress index (Kriticos et al. 2015). The stress threshold and rate for *L. occidentalis* indicate that this species can tolerate a broader climate range than other species, confirmed by its higher niche similarity than other example species (Byeon, Jung, et al. 2021). Significant differences were observed when comparing the predicted distributions of three major pests causing extensive damage to agriculture, based on existing climate databases and updated climate databases. This suggests that the importance of continuously updating climate databases will increase in order to reflect the climate characteristics of each region over time and changes in species distribution areas due to climate change when species distribution modeling.

Despite the advantages of the updated climate database, several limitations should be considered. Relative humidity was not included because globally consistent monthly humidity data covering the study period were not available from the public climate source used to construct the database. Although humidity can influence certain CLIMEX stress indices for some taxa, temperature and precipitation are generally regarded as the primary climatic drivers of insect distributions and were available with consistent global coverage. Therefore, the omission of humidity represents a limitation of the present database and may introduce additional uncertainty for species that are particularly sensitive to atmospheric moisture. In addition, the database was developed at a spatial resolution of 18.5 km, which was intended primarily for global and regional‐scale species distribution modeling and pest risk assessment. Although higher‐resolution climate datasets may be preferable for local‐scale habitat analyses, global‐scale studies commonly employ coarser climate layers because they provide a practical balance between spatial detail, global coverage, and computational efficiency (Cornelissen et al. 2019; Byeon and Lee 2022; Yoon et al. 2026). The selected resolution was sufficient for identifying broad‐scale patterns of climatic suitability and potential invasion risk, but may not fully capture microclimatic refugia, local topographic variation, or fine‐scale habitat heterogeneity. Although interpolation techniques can be used to derive higher‐resolution climate surfaces from lower‐resolution datasets for local‐scale applications, the resulting predictions may be subject to additional uncertainty (Byeon and Lee 2025). Therefore, caution is warranted when interpreting predictions at local scales. Future database updates incorporating globally consistent humidity datasets and the development of high‐resolution regional databases may further improve the accuracy and applicability of species distribution modeling across different spatial scales. Although TSS provides a practical common metric for SDM and is potentially applicable for either mechanistic or correlative SDMs (Yoon and Lee 2023), its value may vary depending on threshold selection and the prevalence structure of occurrence data. Therefore, model performance should be interpreted together with complementary metrics, including sensitivity, specificity, AUC, and recall, rather than relying solely on a single threshold‐dependent measure.

The findings of this study underscore the transformative impact that accessible, high‐quality climate services can have on agricultural and forestry biosecurity policies. Although the overall predicted distributions showed high niche similarity, the updated climate database revealed localized shifts in climatic suitability within potential distribution areas. Areas previously classified as low risk under historical climate baselines were reclassified as moderate or high risk under the 1992–2021 climate database. From an operational biosecurity perspective, these fine‐scale spatial differences may influence the placement of early‐warning monitoring programs and the delineation of quarantine management zones. By providing a temporally synchronized climate database, this service allows policymakers to base these high‐stakes economic and ecological decisions on contemporary environmental realities rather than obsolete baselines. This shift mitigates the risk of ‘false negatives’ in policy—where emerging vulnerable regions are left unmonitored—thereby optimizing the allocation of limited public funding for pest management. Furthermore, a significant contribution of this work lies in identifying and overcoming the structural barriers to the effective integration of climate knowledge. The primary bottleneck for practitioners is rarely a lack of theoretical understanding, but rather the immense technical friction involved in transforming raw climatological data into standardized, biologically relevant formats. By releasing this validated, updated database as an open‐access, ready‐to‐use service, we bypass the computational and disciplinary barriers that typically isolate climate science from applied operational ecology. The open accessibility of this database allows agricultural authorities in developing nations with limited resources to incorporate contemporary climate services into their biosecurity and quarantine frameworks, supporting a more proactive approach to managing invasive species under rapid climate change.

## 5 Conclusion

Updated climate databases are essential to develop models that reflect recent climate and distribution trends. Even with high niche similarity, there are fluctuations in suitability and probability, and the impact of climate data change varies between species. Therefore, as new distribution data are added, updating the climate data available for species distribution modeling is necessary. While there is ongoing work to create high‐resolution regional databases and to develop recently introduced shared socioeconomic pathway climate change scenarios suitable for CLIMEX, our efforts focused on constructing a readily accessible climate database for species distribution modeling and validating its applicability using representative example species. This study makes a contribution by establishing a contemporary climate database compatible with CLIMEX, for which readily accessible and up‐to‐date datasets have been lacking, while simultaneously generating bioclimatic variables for correlative species distribution models. By supporting both mechanistic and correlative modeling approaches, these resources enable the development of SDMs that more accurately reflect contemporary distribution and climatic trends.

## Author Contributions

**Jae‐Woo Song:** conceptualization (equal), data curation (lead), formal analysis (lead), investigation (lead), methodology (lead), software (lead), validation (lead), visualization (lead), writing – original draft (lead), writing – review and editing (equal). **Sunhee Yoon:** formal analysis (supporting), investigation (equal), methodology (supporting), software (equal). **Sunghoon Jung:** funding acquisition (lead), project administration (lead), supervision (supporting). **Wang‐Hee Lee:** conceptualization (lead), investigation (supporting), methodology (supporting), supervision (lead), writing – original draft (supporting), writing – review and editing (lead).

## Funding

This work was supported by Rural Development Administration (RS‐2025‐02214176).

## Conflicts of Interest

The authors declare no conflicts of interest.

## Supporting information


**Code S1.** Python script used to convert the processed climate‐data CSV file into CLIMEX (MetManager)‐compatible .met and .loc files for construction of the meteorological database.

## Data Availability

The climate databases generated during the current study are available for download from Zenodo at https://doi.org/10.5281/zenodo.15392826.
