# Supplementary material for: Impact of an Updated Climate Database on Model Performance and Niche Variability in Species Distribution Modeling
Source: Ecol Evol. 2026 Jun 23;16(6):e73891. doi: 10.1002/ece3.73891 (PMC13288864; doi:10.1002/ece3.73891)
Supplement: Supplementary file 1 — Code S1. Python script used to convert the processed climate‐data CSV file into CLIMEX (MetManager)‐compatible .met and .loc files for construction of the meteorological database. [file ECE3-16-e73891-s001.docx]

**Code S1.** Python script used to convert the processed climate-data CSV file into CLIMEX (MetManager)-compatible .met and .loc files for construction of the meteorological database.

import pandas as pd

import numpy as np

from tqdm import notebook

import time

df=pd.read_csv("D:/bio/worldclim_1991-2021/world2021_F.csv")

df

save_loc1='World_ssp245_10m_1_F.loc'

save_loc2='World_ssp245_10m_2_F.loc'

save_met1='World_ssp245_10m_1_F.met'

save_met2='World_ssp245_10m_2_F.met'

save_loc1='World_ssp585_10m_1_F.loc'

save_loc2='World_ssp585_10m_2_F.loc'

save_met1='World_ssp585_10m_1_F.met'

save_met2='World_ssp585_10m_2_F.met'

cont='World'

count='nocount'

state='nostates'

locfile1="C:/Users/Owner/Workspace/World_ssp585_10m_1_F.loc"

locfile2="C:/Users/Owner/Workspace/World_ssp585_10m_2_F.loc"

lon=df[['longitude']]

lat=df[['latitude']]

latlon= df["latitude"].apply(str) + "N_" + df["longitude"].apply(str) + "E"

df=df.drop_duplicates(['longitude', 'latitude'], keep='first')

zero = []

for i in range(len(lon)):

zero.append(0)

zero=pd.DataFrame(zero)

zero.head()

loc=pd.concat([lat,lon,zero,latlon,zero],axis=1)

loc.columns = ['latitude', 'longitude','zero','location','zero']

loc.head()

tma=df[['tmax1','tmax2','tmax3','tmax4','tmax5','tmax6','tmax7','tmax8','tmax9','tmax10','tmax11','tmax12']]

tmi=df[['tmin1','tmin2','tmin3','tmin4','tmin5','tmin6','tmin7','tmin8','tmin9','tmin10','tmin11','tmin12']]

pre=df[['prec1','prec2','prec3','prec4','prec5','prec6','prec7','prec8','prec9','prec10','prec11','prec12']]

tma.head()

tmax=np.array(tma)

tmin=np.array(tmi)

prec=np.array(pre)

mo=1,2,3,4,5,6,7,8,9,10,11,12

climex_F= pd.DataFrame(index=range(0,len(df)*12), columns = ['month', 'tmax','tmin','prec','am9','pm3','location'])

climex_F=np.array(climex_F)

climex_F

for j in notebook.tqdm(range(0,len(df))):

for i in range(0,12):

z=i+(12*(j))

climex_F[z]=np.array([mo[i], tmax[j,i], tmin[j,i], prec[j,i],"0","0",latlon[j]])

climex_F=pd.DataFrame(climex_F)

climex_F.columns = ['month', 'tmax','tmin','prec','am9','pm3','location']

climex_F

loc_F1=loc.iloc[0:480000]

loc_F2=loc.iloc[480000:len(loc)]

climex_met1=climex_F.iloc[0:5760000]

climex_met2=climex_F.iloc[5760000:len(loc)*12]

climex_met1.to_csv(save_met1, index=False, header=False, sep=',')

climex_met2.to_csv(save_met2, index=False, header=False, sep=',')

options=[['#Continent',cont,'','',''], ['#Country',count,'','',''], ['#State', state,'','','']]

options=pd.DataFrame(options,columns=['latitude', 'longitude','zero','location','zero'])

climex_loc1=pd.concat([options, loc_F1])

climex_loc2=pd.concat([options, loc_F2])

climex_loc1.to_csv(save_loc1, index=False, header=False, sep=',')

climex_loc2.to_csv(save_loc2, index=False, header=False, sep=',')

conta='#Continent,'+cont+',,,\n'

contb='#Continent, '+cont+'\n'

counta='#Country,'+count+',,,\n'

countb='#Country, '+count+'\n'

statea='#State,'+state+',,,\n'

stateb='#State, '+state+'\n'

with open(locfile1,"rt") as f:

lines1 = f.read()

with open(locfile1, "wt") as f:

lines1=lines1.replace(conta,contb)

lines1=lines1.replace(counta,countb)

lines1=lines1.replace(statea,stateb)

f.write(lines1)

with open(locfile2,"rt") as f:

lines2 = f.read()

with open(locfile2, "wt") as f:

lines2=lines2.replace(conta,contb)

lines2=lines2.replace(counta,countb)

lines2=lines2.replace(statea,stateb)

f.write(lines2)
